# Ramadan Fasting Exerts Immunomodulatory Effects: Insights from a Systematic Review

**DOI:** 10.3389/fimmu.2017.01144

**Published:** 2017-11-27

**Authors:** Mohammad Adawi, Abdulla Watad, Stav Brown, Khadija Aazza, Hicham Aazza, Mohamed Zouhir, Kassem Sharif, Khaled Ghanayem, Raymond Farah, Hussein Mahagna, Stefano Fiordoro, Samir Giuseppe Sukkar, Nicola Luigi Bragazzi, Naim Mahroum

**Affiliations:** ^1^Padeh and Ziv Hospitals, Bar-Ilan Faculty of Medicine, Zefat, Israel; ^2^Department of Medicine ‘B’, The Zabludowicz Center for Autoimmune Diseases, Sheba Medical Center, Tel-Hashomer, Israel; ^3^Sackler Faculty of Medicine, Tel-Aviv University, Tel-Aviv, Israel; ^4^Faculty of Sciences Dhar Mahraz, Sidi Mohamed Ben Abdellah University, Fez, Morocco; ^5^Faculty of Literature and Humanistic Studies, Sidi Mohamed Ben Abdellah University, Fez, Morocco; ^6^Department of Internal Medicine, Ziv Medical Center, Safed, Israel; ^7^Immunology Service, Ospedale Policlinico San Martino, Genoa, Italy; ^8^Clinical Nutrition Unit, Ospedale Policlinico San Martino, Genoa, Italy; ^9^Department of Health Sciences (DISSAL), School of Public Health, University of Genoa, Genoa, Italy

**Keywords:** Ramadan, fasting, immune system, autoimmunity, antibodies

## Abstract

Ramadan is the ninth month of the Islamic lunar calendar and is observed by Muslims as a month of fasting. All Muslim adults are expected to fast; nevertheless certain subgroups, including sick, frail subjects, and pregnant women, among others, are exempted. Ramadan fasting has been shown to impact on body systems in different manners. The influence of Ramadan fasting on immune system regulation remains elusive; however, immune system changes, such as the modulation of body response to various infectious, stressful, and other harmful events, are of great interest during fasting. In this paper, we performed an extensive systematic literature review of different scholarly databases (ISI/Web of Science, Scopus, PubMed,/MEDLINE, Google Scholar, Directory of Open Access Journals, EbscoHOST, Scirus, Science Direct, the Cochrane Library, and ProQuest), using the following key words: “fasting,” “Ramadan,” “Islam,” and “immunity.” Conclusions drawn from these findings included: (1) Ramadan fasting has been shown to only mildly influence the immune system and the alterations induced are transient, returning to basal pre-Ramadan status shortly afterward. (2) Ramadan fasting during the second trimester of pregnancy was shown to be safe and did not result in negative fetal outcomes, or maternal oxidative status alterations. (3) In cardiac patients, Ramadan fasting can have beneficial effects including lipid profile improvement and alleviation of oxidative stress. (4) In asthmatic patients as well as in patients with human immunodeficiency virus/acquired immunodeficiency syndrome and autoimmune disorders, fasting was safe. (5) In psychiatric patients, such as those suffering from schizophrenia, fasting could increase immunologic markers. (6) Fasting Muslim athletes who maintain intensive training schedule during Ramadan showed fluctuations of immunologic markers.

## Introduction

Ramadan, the ninth month of the Islamic lunar calendar, is observed by approximately 1.8 billion Muslims worldwide as a month of fasting and as one of the five pillars of Islam, along with testimony of faith, charity, pilgrimage, and daily prayer. It is believed that the revelation of the *Qu’ran* to Prophet Muhammad occurred during this month.

During daylight hours, Muslims abstain from eating any food, drinking any liquid, and other physical needs such as smoking and sexual intercourse. Furthermore, Ramadan has a strong ethical and spiritual dimension, in that it is a time to purify the soul and to gain proximity to God (“*Allah”*) through reflection and supplication. While Muslims fast from dawn until sunset, food and drinks are allowed before dawn and after sunset (these meals are called *Suhoor* and *Iftar*, respectively) (1). The month of Ramadan lasts 29–30 days based on the witnessing of the small sliver of the crescent moon. The duration of the month varies upon the time of year and regional latitudes. Mean fasting duration is usually 13 h, whereas in some regions it can extend to 18 h (1).

All Muslim adults are expected to fast; nevertheless, there exist dispensations for certain subsets of the Islamic community including pregnant, nursing or menstruating women, travelers, ill people, and the very young or the very old individuals.

As Ramadan fasting influences different body systems, of great interest is the influence of fasting on the immune system, which plays a critical role in regulating and maintaining body response to stressful and harmful events. This influence may shed light on immune status of fasting people. The clinical relevance of such topic stems from the fact that, in the world of globalization, any physician could face the issue of managing patients who choose to fast Ramadan and monitoring their immune status (2, 3). However, no guidelines or protocols exist that provide guidance toward a patient-tailored approach.

The purpose of this review was to fill this gap in knowledge, providing an insight on the possible influence of Ramadan fasting on the immune system.

## Materials and Methods

A systematic review according to the “Preferred Reporting Items for Systematic Reviews and Meta-Analyses” guidelines was performed by searching different databases, including ISI/Web of Science, Scopus, PubMed/MEDLINE, Directory of Open Access Journals, EbscoHOST, Scirus, Science Direct, the Cochrane Library and ProQuest. Search was carried out using a string made up of combination of key words including “Islam,” “Ramadan,” “fasting,” and “immunity.”

Gray literature was also manually searched *via* Google Scholar. Review articles or research manuscripts not pertinent with the aim of this systematic review were excluded, while all the other research articles (including editorials, letters, case reports) were retained if containing sufficient quantitative details. Neither time nor language filters were applied.

Review Manager version 5.3 (RevMan5.3, Cochrane) was used for handling all the processes of the present systematic review.

**Figure 1:**
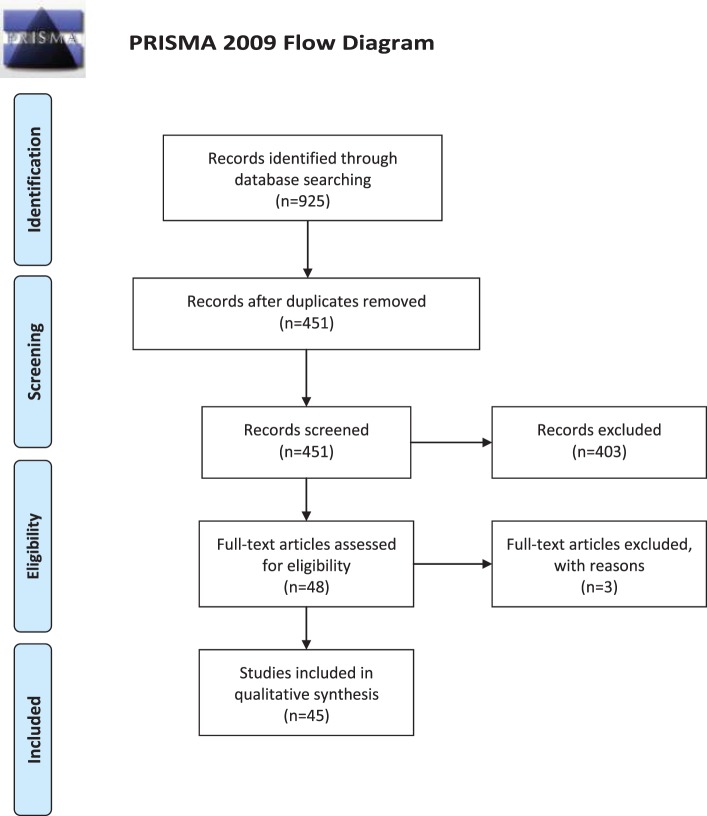
Preferred Reporting Items for Systematic Reviews and Meta-Analyses 2009 Flow Diagram.

For further details, the reader is referred to Table 1 and Figure 1.

**Table 1:** Search strategy details.

Search strategy item	Search strategy details
Used keywords	(“Ramadan fast” OR “Ramadan fasting” OR “Ramadan month”) AND (immune OR immunity OR immunologic OR lymphocyte OR chemokine OR interleukin OR C-reactive protein OR CRP OR neutrophils OR “oxidative stress” OR “oxidative burst” OR inflammatory OR inflammation OR immunoglobulin OR autoimmune OR “lipid peroxidation” OR homocysteine OR malondialdehyde OR MDA OR glutathione OR GSH)

Searched databases	PubMed/MEDLINE, Scirus, Scopus, Directory of Open Access Journals, Google Scholar, ISI/Web of Science

Inclusion criteria	P: muslim subjects
	I: subjects willing to fast during the month of Ramadan
	C: subjects fasting *versus* subjects not fasting during the month of Ramadan
	O: impact of Ramadan fasting on immune system
	S: original article

Exclusion criteria	Studies not reporting the impact of Ramadan fasting on immune system
	Letter to editor, editorial, expert opinion, commentary not containing sufficient quantitative details, review article

Time filter	None applied (from inception)

Language filter	None applied (in any language)

Target journals	Allergologia et immunopathologia; Annals of Nutrition and Metabolism; Applied Physiology, Nutrition, and Metabolism; Asian Journal of Sports Medicine; Blood Pressure; European Journal of Nutrition; European Journal of Pediatrics; Indian Journal of Gastroenterology; International Journal of Pharmaceutical Science Invention; International Journal of Sport Nutrition and Exercise Metabolism; Iranian Journal of Basic Medical Sciences; Journal of Ayub Medical College, Abbottabad; Journal of Biological Sciences; Metabolic Syndrome and Related Disorders; Nutrition Journal; Nutrition Research; PLoS ONE; Psychiatry Research; Rheumatology International; The Journal of International Medical Research; Therapeutic Advances in Endocrinology and Metabolism; Transplantation Proceedings; Tropical Doctor; Vascular Health and Risk Management; World Journal of Medical Sciences

## Results

The initial search in databases yielded a pool of 925 items. After removing duplicates and not pertinent studies, as well as excluding studies with reasons (Table 2), 45 studies were included in the current systematic review (Table 3). Twenty-five studies focused on the impact of fasting on immune system in healthy subjects, while five investigated the impact of Ramadan on immune system in people suffering from autoimmune diseases (such as systemic lupus erythematosus or SLE, multiple sclerosis or MS, and inflammatory bowel disease or IBD). Three articles explored the effect of fast on immune system in patients with cardiac diseases, whereas one and eight investigations assessed the influence of fasting in pregnant women and athletes, respectively. One study was conducted among people with human immunodeficiency virus (HIV)/acquired immunodeficiency syndrome, while another one was devoted to describe the impact of Ramadan in patients with psychiatric disorders (schizophrenia). The remaining study recruited asthmatic people.

**Table 2:** Studies excluded with reason.

Excluded study with reason	Reason for exclusion
Razeghi Jahromi et al. (4)	Animal model
Shawky et al. (5)	Animal model
Sadek and Saleh (6)	Animal model

**Table 3:** Studies on Ramadan Fasting and the Immunity System stratified by different topics.

Topic	Immunologic/inflammatory markers studied		Reference	Country	Study design	Sample timing	*N*	Mean age/age range (years)	Gender	Conclusion
Immune system and healthy subjects	hs-CRP, GGT, DUSP1, and interleukin 1α (IL-1α) gene expression in circulating leukocytes		(7)	Saudi Arabia	PCS	BR, 10–15 d DR	23	23.2 ± 1.2 (18–42)	M (18) + F (5)	Mean level of GGT decreased during the fast. Mean morning and evening DUSP1 level significantly increased during the fast, even though diurnal rhythm was preserved. Morning IL-1α level was higher than in the evening; however, mean value decreased during the fast with respect to the period before the fast. Diurnal rhythm of hs-CRP was lost

	IL-6, hs-CRP		(8)	Iran	PCS	1 w BR, 4 w DR, 4 w AR	30	29.44 ± 7.4 (20–35)	M	No significant changes could be detected

	WBC count and tuberculin induration		(9)	Iran	PCS	4 w DR, 3 m AR	28	19.21 ± 3.83 (14–35)	M	Neutrophil count and lymphocyte count were significantly reduced, no association between PPD test and Ramadan fasting was noted

	Lipid peroxidation profile, MDA		(10)	Saudi Arabia	PCS	BR, DR, AR	8	26.6 ± 4.9 (20–35)	M	No significant changes could be detected

	IgG, IgA, IgM levels		(11)	Saudi Arabia	PCS	BR, 2 w DR	23	23.2 ± 1.2 (18–42)	M (18) + F (5)	Marked decrease in IgG level

	Pro-oxidant and antioxidant profiles		(12)	Iran	PCS	1 w BR, 27 d DR	23	25–65	M (16) + F (7)	No significant changes

	CRP, granulysin levels		(13)	Iran	PCS	29 d DR, 4 m AR	44	41.15 ± 13.6	M	Statistically significant decrease in CRP, no differences in granulysin level

	TNF-α		(14)	Iran	PCS	1–2 d DR, last 3 d of fasting	70	47.88 (30–70)	M	No changes in TNF-α

	C3, iNOS and SOD levels in serum and the killing ability of PBMC against *Mycobacterium tuberculosis*		(15)	Indonesia	PCS	7 d BR, 7 d DR, 21 d DR	30	20.26 ± 1.13 (18–22)	M	Ramadan fasting was shown to enhance the killing ability of macrophages against *M. tuberculosis*. Ramadan fasting did not alter the serum levels of complement C3, iNOS, or SOD

	CXCL1, CXCL10, and CXCL12 chemokines levels		(16)	Iran	PCS	BR, AR	58	20–40	M	Significant decrease in BWC, pro-inflammatory chemokines (CXCL1, CXCL10), and the constitutive chemokine (CXCL12), TNF-α,IL-2, IL-8—fasting plays a role in controlling inflammation *via* chemokines

	Serum IgG and IgM levels, salivary IgA levels		(17)	Turkey	PCS	BR, 25 d DR	35	35.86 ± 11.07 (20–59)	M	Ramadan fasting was not shown to result in severe immunological disturbances. However, even though remaining in the normal range, IgG and IgA decreased significantly, IgM did not change. Lymphocyte number increased. No correlation between immunoglobulin levels and lymphocyte number could be found

	Serum, PBMC and macrophages endorphin and endocannabinoid levels		(18)	Indonesia	PCS	1 w BR, 1 w DR, and 3 w DR	27	20.26 ± 1.13 (18–22)	M	Endorphin levels were significantly elevated in serum, PBMC, and macrophages during Ramadan as compared to before. Additionally, endocannabinoid levels were significantly elevated in serum and PBMC. In contrast, endocannabinoid levels were noted to be significantly low in macrophages. The impact of Ramadan fasting on these molecules appears to be subtle

	IFN-γ, TNF-α, iNOS, and SOD		(19)	Indonesia	PCS	1 w BR, 1 w DR, and 3 w DR	27	20.26 ± 1.13 (18–22)	M	Significant increase of IFN-γ, TNF-α, and iNOS, significant decrease of SOD- Ramadan fasting induces activation and inflammation while decreasing oxidative stress on macrophages

	Urinary 15FIP levels		(20)	Jordan	PCS	1 w BR, 22 d DR, 30 d AR	50	18–51	M (23) + F (27)	Levels of urinary 15FIP were significantly elevated

	Circulating pro-inflammatory cytokines (IL-1β, IL-6, and TNF-α), immune cells (total leukocytes, monocytes, granulocytes, and lymphocytes)		(21)	Jordan	PCS	1 w BR, 3 w DR, 1 m AR	50	18–51	M (21) + F (29)	Statistically significant decrease during Ramadan with respect to the period before and after. Ramadan induces immune attenuation

	IL-1α, IL-2, IL-6, and IL-8		(22)	Iraq	Case–control study	n.a.	30 cases and 30 controls	34.5 ± 11.5 (21–58)	n.a.	Ramadan may induce immunomodulation but changes in interleukins did not reach statistical significance

	MDA and GSH levels		(23)	Turkey	PCS	BR, 28 d DR/AR	45	28.7 (21–51)	M (23) + F (22)	MDA levels increased in both genders; however, the increase was statistically significant only in female subjects. GSH levels decreased in males, however, increased in females—fasting may modify the immune response by inducing oxidative stress, having a gender-specific effect

	Neutrophil phagocytosis, serum opsonization power, and NBT reduction		(24)	Iran	PCS	BR, AR	13	28–54	M	Ramadan fasting had a beneficial effect on neutrophils phagocytic function

	MDA, GSH, glutathione peroxidase, and catalase levels		(25)	United Arab Emirates	PCS	2 d BR, 14 d DR, 28 d DR	14	25–58	M (9) + F (5)	Ramadan fasting was not associated with alterations in parameters that reflect oxidative stress or biochemical markers

	Neutrophil activity		(26)	Iran	PCS	BR, AR	24	26.5 (18–35)	M	No significant changes in neutrophil activity

	Homocysteine, CRP, and IL-6 levels		(27)	Turkey	Case–control study	1 w BR, 4 w DR, 20 d AR	40 cases and 28 controls	20–40	M (34) + F (34)	Statistically significant decrease in IL-6, CRP, homocysteine levels

	CIC levels		(28)	Iran	PCS	BR, AR	28	26.2	M	No significant changes in CIC levels

	CL activity and CIC levels		(29)	Iran	PCS	BR, AR	21	26.5 (18–35)	M	No significant differences in CL activity and CIC levels

	C3, C4, CHSO		(30)	Iran	PCS	BR, AR	50	20–25	M + F	No significant changes

	MDA, conjugated dienes		(31)	Iran	PCS	BR, AR	46	30–60	M	Statistically significant decrease in MDA, increase in conjugated dienes induced by the Ramadan fasting

Immune system and autoimmune diseases	SLE	Levels of autoantibodies	(32)	Iran	Case–control study	BR/DR, AR	21 cases and 19 controls (out of 80 eligible patients, 38 cases and 42 controls)	39.7 ± 13.4 for cases, 40.2 ± 11.2 for controls	F	Anti-dsDNA and C3 levels significantly increased levels. No changes in disease activity or quality of life were noted—Ramadan fasting has minimal and non-detrimental effects of on SLE patients

	MS	Antioxidant status	(33)	Egypt	Case–control study	BR, DR, AR (1 year of follow-up)	15 cases, 15 controls	15–45	n.a.	No significant differences in relapse rate, EDSS score, or contrast enhanced lesions on MRI—Ramadan fasting did not pose any unfavorable outcomes in MS patients. On the contrary, Ramadan could improve antioxidative status and protect against relapse, by reducing levels of uric acid

	Rheumatoid arthritis (RA)	CIC	(34)	India	PCS	BR, AR	30	45–60	M (5) + F (25)	Ramadan could be beneficial for RA patients

	MS	Frequency of relapse rate and EDSS score	(35)	Iran	Case–control study	DR	80 (40 cases, 40 controls)	28.73 ± 6.80 for cases, 31.10 ± 9.09 for controls	M (29) + F (51)	No significant differences in relapse rate or EDSS score—Ramadan fasting did not pose any unfavorable outcomes in MS patients

	IBD	Disease severity and quality of life	(36)	Iran	PCS	BR, AR	60	35.5 ± 15	M (27) + F (33)	No correlation between fasting, disease severity, or quality of life was documented. This result highlights the safety of Ramadan fasting in IBD patients

Immune system and asthmatic patients	WBC, hs-CRP		(37)	Iran	Case–control study	3–5 d BR, AR	29 (15 cases and 14 controls)	49.28 ± 12.54 (cases), 37.5 ± 7.86 (controls)	n.a.	Decrease in hs-CRP

Immune system and cardiac diseases	PP, MDA, and GSH levels		(38)	Egypt	n/a	From 1 w BR to 6 w AR	n/a	55 ± 5	n.a.	Significant reduction of PP and MDA and significant increase in GSH
	hs-CRP levels		(39)	Qatar	PCS	BR/DR, AR	56	18 ≤ 50 y, 38 > 50 y	M (45) + F (11)	No cardiac or non-cardiac morbidity or mortality was reported. Biochemical levels of hs-CRP were not shown to significantly vary—Ramadan fasting is safe in cardiac patients with stable cardiac conditions
	hs-CRP and homocysteine levels, complete blood count		(40)	Iran	PCS	7 d BR, 1 d DR, 2 d DR, from 27 d DR to 6 AR	82	54.0 ± 10.0 (29–70)	M (38) + F (44)	Significant improvement of the 10-year coronary heart disease risk. No significant alterations were found in hs-CRP and homocysteine levels—Ramadan fasting improves CVD risk factors

Immune system and human immunodeficiency virus patients	Cluster differentiation 4 (CD4) cell count, viral load		(41)	Nigeria	PCS	BR, DR, AR	17	n.a.	n.a.	No changes in CD4 cell count, viral load, and hematocrit could be detected

Immune system and pregnant women	Serum TAS, TOS, and OSI		(42)	Turkey	Case–control study	n.a.	42 cases and (48 potentially eligible) 30 controls	30.10 (20–43) for cases, 29.50 (18–46) for controls	F	Ramadan fasting did not have a significant effect on the maternal oxidative stress, fetal development, maternal complications, or fetal birth weight

Immune system and psychiatric patients	WBC, granulocytes, lymphocytes, monocytes, fibrinogen, and hs-CRP		(43)	Egypt	PCS	BR, 4 w DR	100 out of 134 eligible patients	39.6 ± 13.4with metabolic syndrome, 37.9 ± 13.7 without metabolic syndrome	M	Increase in inflammatory markers in patients with schizophrenia and metabolic disorders

Immune systems and athletes	Soccer players	TAS	(44)	Tunisia	PCS	1 w BR, 2 w DR, 4 w DR	12	17.52 ± 0.2	M	Increased TAS

	Rugby players	IgG, IgM, IgA and IgE, C3, C4, and blood count cells	(45)	Iran	PCS	BR, AR	90	16–36	n.a.	Increase in IgA and C4, decrease in percentage of lymphocytes and neutrophils. Ramadan could protect against exercise-induced/related infections

	Soccer players	Homocysteine and hs-CRP levels	(46)	Tunisia	PCS	1 w BR, 2 w DR, 4 w DR	15	17.3 ± 0.3	M	Higher levels of HDL-C and APO-AI were documented and a decrease of low density lipoprotein, apolipoprotein B, lipoprotein particles, homocysteine, and hs-CRP—fasting and exercise work synergistically to reduce body mass, improve lipid profile and modulate the inflammatory status in athletes

	Recreational bodybuilders	CRP	(47)	Tunisia	Case–control study	2 d BR, 29 d DR	16 (9 cases and 7 controls)	24 ± 3for cases, 26 ± 3 for controls	M	No changes

	Physical education students	IL-12 levels	(48)	Tunisia	PCS	1 w DR, 4 w DR, 1–3 w AR	9	22.1 ± 0.2	M	IL-12 was significantly decreased during Ramadan in comparison to after Ramadan. The fluctuation of IL-12 levels has been attributed to changes in dietary intake as well as sleep pattern alterations

	Elite judo athletes	CRP, IgA, IgG, IgM, homocysteine and leukocyte counts, antitrypsin	(49)	Tunisia	PCS	4–5 d BR, 7 d DR, 15–16 d DR, 28–29 d DR	15	18 ± 1	M	Serum CRP and IgA increased, homocysteine remained relatively unchanged, as well as leukocyte counts

	Middle-distance runners	CRP, IL-6	(50)	France	Case–control study	1 d BR, 7, 21 d DR, 31 d DR	8	25.0 ± 1.3	M	MAV test decreased significantly during Ramadan, while hormone levels did not show any significant deviations. Fatigue levels significantly increased, concomitant with a significant increase of IL-6

	Soccer players	Leukocyte count, CRP	(51)	Tunisia	Case–control study	3 w BR, 2 w DR, 4 w DR, 3 w AR	78 (48 cases and 30 controls)	16–19	M	Decreased leukocytes count and CRP

*15FIP, 15-F(2t)-Isoprostane; Anti-dsDNA, anti double strand DNA; AR, after Ramadan; BR, before Ramadan; C3, complement-3; C4, complement 4; CICs, circulating immune complexes; CL, chemiluminescence; CRP, C-reactive protein; CVD, cardiovascular disease; CXCL1, chemokine ligand 1; CXCL10, chemokine ligand 10; CXCL12, chemokine ligand 12; d, day; DR, during Ramadan; EDSS, expanded disability status scale; F, female; GSH, glutathione; hs-CRP, highly sensitive C-reactive protein; IBD, inflammatory bowel disease, PP, pulse pressure; IFN-γ, interferon γ; IgA, Immunoglobulin A; IgG, Immunoglobulin G; IgM, Immunoglobulin M; IL-1β, interleukin 1β; IL-2, interleukin 2; IL-6, interleukin 6; IL-8, interleukin 8; IL-12, interleukin 12; iNOS, inducible nitric oxide synthase; m, month; M, male; MAV, maximal aerobic velocity; MDA, malondialdehyde; MRI, magnetic resonance imaging; MS, multiple sclerosis; n.a., not available; OSI, oxidative stress index; PBMC, peripheral blood mononuclear cells; PCS, prospective cohort study; PPD, purified protein derivative; SLE, systemic lupus erythematosus; SOD, superoxide dismutase; TAS, total antioxidant status; TNF-α, tumor necrosis factor alpha; TOS, total oxidant status; w, week; WBC, white blood cells; y, year*.

Sample-sizes ranged from 8 to 100 recruited subjects. Eighteen studies were carried out in Iran, six in Tunisia, four in Turkey, three in Indonesia, in Saudi Arabia, and in Egypt, two in Jordan, and one in France, in India, in Iraq, in Nigeria, in Qatar, and in United Arab Emirates. Age ranged from 15 to 70 years. Fasting went from 8 to 17 h, depending on the country and on the year of investigation. Completion rate (subjects who completed the study out of the total sample) ranged from 100.0 to 17.5%. Concerning gender, 23 researches investigated samples made up of only males, whereas 20 studies recruited mixed samples. Only two studies were conducted with female samples. Regarding recruiting and sampling strategies, most studies relied on convenience samples/purposive sampling, whereas only two studies (7, 11) computed *a priori* sample-size power. Ten studies were case–control studies, while the remaining ones were observational prospective cohort studies.

## Immunity Changes in Healthy Individuals

Latifynia et al. (28) investigated the influence of Ramadan fasting on neutrophils respiratory burst and circulating immune complexes (CICs) in 24 healthy male volunteers aged 18–45 years. On chemiluminescence (CL), no statistically significant changes on neutrophil activity were obtained, eliminating hazardous effect of Ramadan fasting on the innate system.

In a closely similar study, levels of CICs were determined using several techniques including polyethylene glycol precipitation method, quantitative CL and circulating immune techniques. Authors investigated a sample of 28 healthy students residing in dormitory of Tehran University. The mean age of the enrolled subjects was 26.2 years. The results of this study showed a mean CIC level of 2.04 ± 1.86 before Ramadan and 2.63 ± 2.1 after Ramadan (*p-value* = 0.05). Such finding highlighted the absence of significant changes of CIC levels before and after Ramadan fasting. Of note, CIC levels increased in 17 subjects, whereas in 11 subjects the levels of CIC decreased. Of the enrolled subjects, only six had levels that did not lie within the normal range. In addition, three cases had low CIC levels before Ramadan and remained abnormal thereafter (28). Similar results were replicated in additional studies: CIC levels as well as complement-3 (C3), C4 levels were not demonstrated to be significantly altered as a result of Ramadan fasting (29, 30).

In an additional research, the neutrophil respiratory burst was probed in a small sample of 21 healthy male students from Tehran aged 18–25 years. In 52% of the cases, CL activity and CIC levels pre and post Ramadan remained within the normal range and some patients had an insignificant decrease or increase of CL and CIC levels. In 24% of the cases, CL and CIC levels were measured to be high before Ramadan, with normalization of both parameters after the month of Ramadan. In an additional 24% of the patients, an elevation of both CL and CIC levels was witnessed after Ramadan fasting. Based on the lack of significant differences in CL and CIC levels before and after Ramadan, Ramadan fasting was not shown to influence neutrophil respiratory burst (26).

The intermittent fasting approach has been hypothesized to positively affect the inflammatory state and has been shown to reduce inflammation and prevent cancer promotion in animal models. In humans, a cross sectional study of 50 healthy volunteers (21 men and 29 women) was conducted to explore the fluctuations of circulating pro-inflammatory cytokines (interleukin 1β or IL-1β, interleukin 6 or IL-6, tumor necrosis factor alpha or TNF-α), immune cells (total leukocytes, monocytes, granulocytes, and lymphocytes) at three instances: 1 week before Ramadan, 3 weeks after Ramadan, and 1 month after the conclusion of Ramadan. The pro-inflammatory cytokines IL-1β, IL-6, and TNF-α showed a significant decrease during Ramadan (*p*-value < 0.05). In addition, immune cells significantly dropped during Ramadan, albeit remaining within the reference ranges (21). In another case–control study including 30 fasting patients and 30 non-fasters, interleukin 1α, interleukin 2, IL-6, and interleukin 8 were shown to be decreased although not reaching a significant level, which seems to suggest a possible immunomodulatory influence of Ramadan fasting (22). In a prospective cohort study, TNF-α levels were not shown to be changed post fasting (14). Taken together, these results support the perception of immune attenuation during Ramadan.

The role of Ramadan fasting on the levels of immunoglobulins was explored by Develioglu et al. (17) who collected blood and saliva samples of 35 healthy male volunteers (mean age of 38 years). The samples were collected 1 week before and during the first week of Ramadan. Immunoglobulin G (IgG) and immunoglobulin A (IgA) concentrations decreased significantly during Ramadan, however, still remained within the normal range. In comparison, the levels of immunoglobulin M remained stable, with no significant increase or decrease. In an additional study, only levels of IgG were shown to be markedly decreased (11). Taken together, the results highlighted that Ramadan fasting was not shown to result in drastic disturbances.

Akrami Mohajeri et al. (16) studied chemokine (C–X–C motif) ligand 1 (CXCL1), chemokine (C–X–C motif) ligand 10 (CXCL10) and chemokine (C–X–C motif) ligand 12 (CXCL12) chemokines levels using enzyme-linked immunosorbent assay (ELISA), in a sample of 58 healthy fasting subjects, aged 20–40 years. Their results showed a significant decrease of the pro-inflammatory chemokines (CXCL1, CXCL10) and the constitutive chemokine (CXCL12). These chemokines are known to be downstream targets of TNF-α, which has been shown to be downregulated during fasting (21), even though some other studies found that TNF-α was increased (19) or remained unchanged (14). Thus, fasting has been shown to play a role in controlling inflammation *via* chemokines.

In another report, 40 healthy fasting volunteers of normal weight (20 females aged 20–38 years and 20 males aged 23–39 years) were recruited and compared to 28 healthy, age- and body mass index (BMI)-matched non-fasting volunteers (14 males, 14 females). Biochemical parameters were monitored including C-reactive protein (CRP), IL-6, and homocysteine or hcy levels. The levels of the inflammatory markers (IL-6, CRP, and hcy) were shown to be significantly decreased as a result of Ramadan fasting in both genders as compared to their primary basal values (50). Gorjipour et al. (13) and Ajabnoor et al. (7) showed similar significant decrease of CRP, whereas only one study failed to demonstrate this effect (8).

The influence of Ramadan fasting on complement C3, inducible nitric oxide synthase (iNOS), superoxide dismutase (SOD) levels in serum and peripheral blood mononuclear cells (PBMC) and macrophages in 30 healthy male volunteers was also monitored to determine whether fasting could alter the ability of PBMC to kill *Mycobacterium tuberculosis* (*M. tuberculosis*). Ramadan fasting was shown to enhance the killing ability of PBMC and macrophages against *M. tuberculosis* when compared to serum activity. There was no alteration on the serum levels of complement C3, iNOS, or SOD during Ramadan fasting (15). Furthermore, Ramadan fasting in a sample of 30 male patients was shown to enhance macrophagic killing abilities against tuberculosis (15).

The influence of Ramadan fasting on tuberculin skin test or purified protein derivative PPD, which is a delayed type hypersensitivity immune response test, has also been addressed for the possible role of Ramadan in immunomodulation. Twenty-eight healthy males were monitored for their white blood cell count and tuberculin induration on the fourth week of Ramadan and 3 months after Ramadan. It was found that while neutrophil and lymphocyte counts were significantly reduced, no association between PPD test and Ramadan fasting was noted (9). In healthy individuals, neutrophil phagocytic function was shown to be improved (24).

From an immunologic point of view, Ramadan fasting could be perceived as a stressor leading to alteration of immune system. Macrophage is usually stimulated to secrete myriad of molecules and chemokines in response to stress and oxidative damage. Lahdimawan et al. (19) investigated the functions of macrophage activity in a sample of 27 male healthy volunteers aged 18–22 years who fasted Ramadan. Significant increases of interferon-gamma, TNF-alpha, and iNOS were noted, while SOD decreased significantly. The results of this study suggest that Ramadan fasting induces activation and inflammation while decreasing oxidative stress on macrophages.

The role of Ramadan fasting as immunologic stressor intrigued researchers to study its impact on endorphin and endocannabinoid levels. Twenty-seven healthy male volunteers aged 18–22 years were enrolled in the study. Blood samples were analyzed using ELISA method for endorphin and endocannbinoid in serum, PBMC, and macrophages. Endorphin levels were noted to be significantly elevated in serum, PBMC and macrophages during Ramadan as compared to before. Additionally, endocannabinoid levels were significantly elevated in serum and PBMC. In contrast, endocannabinoid levels were noted to be significantly low in macrophages. The impact of Ramadan fasting on these molecules appears to be subtle. Endorphins and endocannabinoids share an integral role of immune system regulation by inhibition of T helper cell function and downregulation of antibody production (18).

The influence of Ramadan fasting on the markers of oxidative stress and the biochemical markers of cellular damage in healthy subjects was explored in 14 healthy volunteers, nine men and five women, aged 25–58 years. In these subjects, malondialdehyde (MDA), glutathione (GSH), GSH peroxidase, and catalase levels were monitored. The researchers concluded that, besides the mild reduction of lipid peroxidase levels, Ramadan fasting was not associated with alterations in parameters that reflect oxidative stress or biochemical markers (25). These results were replicated in additional studies that showed a lack of significant change in oxidative stress after Ramadan fasting (10, 12, 31).

Isoprostans, which are prostaglandin F2 like compounds, possess high sensitivity and specificity to oxidative stress. The measurement of prostaglandin F2 has been shown to provide insight to the oxidative stress in the patient’s body. The impact of Ramadan fasting on the oxidative stress was measured in a sample of 50 healthy subjects (23 men and 27 women) by probing the levels of the 15-F(t2)-Isoprostane (15FIP). The levels of urinary 15FIP was significantly elevated when measured in the third week of Ramadan, a finding that was concomitant with significant increase of the body weight and total body fat present. The increase of 15FIP is viewed as reflection of compensatory mechanism known as metabolic adaptation theory, which serves the purpose of opposing further weight change. Increased fat percentage and weight gain (which usually occur in Ramadan) are associated with increased fat oxidation, which leads to increased levels of 15FIP (20).

While the majority of the studies showed a rather neutral effect of Ramadan fasting on the immune system, Sülü et al. (23) investigated MDA and GSH levels in a sample of 45 healthy volunteers (22 females, 23 males, aged 21–51 years, mean age 28.7 years). MDA levels increased in both genders; however, the increase was statistically significant only in female subjects. GSH levels decreased in males, however, increased in females. The authors concluded that the impact of Ramadan fasting on the immune system may be different depending on the socioeconomic status, nutritional habits, and metabolic structure of the people. The same explanation was applied by the authors regarding the induction of oxidative stress that may develop during fasting.

In conclusion, Ramadan’s influence on the immune system still requires further elucidation, yet general trends can be identified. Ramadan fasting has a modulatory effect on chemokine network, oxidative stress and adaptive, and innate immunity.

## Immunity Changes in Autoimmune Diseases

Most of the studies addressing the influence of Ramadan fasting on the immune system have been studied on healthy subjects. Research addressing the impact of Ramadan fasting on the etiopathogenesis as well as the progression and of autoimmune disease is limited. Goharifar et al. (32) conducted a case–control study investigating the impact of Ramadan fasting on SLE disease activity. Forty SLE quiescent patients were enrolled (21 fasting cases, and 19 non-fasting controls). SLE disease activity index, quality of life, and levels of autoantibodies were monitored 1 day before Ramadan, 1 day after Ramadan, and 3 months after Ramadan. Anti-dsDNA is one of the most specific autoantibodies in SLE. This antibody is associated with more severe SLE and with glomerulonephritis. Its level was found to be significantly increased in the fasting group after Ramadan fasting as compared to non-fasters. The levels of Anti-dsDNA remained significantly high after 3 months. Similarly, C3 levels were significantly elevated in the cases versus control group. In contrast, C3 levels dropped to baselines at 3 months. These changes were not accompanied by changes in disease activity or quality of life. These findings highlight the minimal and non-detrimental effects of Ramadan fasting in SLE patients.

The influence of Ramadan fasting on MS disease progression was investigated in two independent studies. The first study, which monitored 30 MS patients with an expanded disability status scale (EDSS) score <3, failed to reveal any significant differences in relapse rate, EDSS score or contrast enhanced lesions on magnetic resonance imaging of the patients who fasted versus the non-fasters (33). Likewise, an additional study on 80 MS patients with EDSS score of <3 did not show any significant increase in the frequency of relapse rate or EDSS score changes at 6 months follow-up (35). Therefore, Ramadan fasting did not pose any unfavorable outcomes in MS patients.

Tavakkoli et al. (36) conducted a cohort study to investigate the impact of Ramadan fasting on IBD progression. In 60 investigated patients, no correlation between fasting, disease severity, or quality of life was documented. This results highlight the safety of Ramadan fasting in IBD patients.

The influence of Ramadan fasting on other autoimmune disease has not been investigated so far. In rheumatoid arthritis (RA), Sharma et al. (34) showed a decrease in CIC levels in 70% of patients after fasting in comparison to pre-Ramadan levels, which suggests a possible beneficial role of fasting in RA patients. In murine models, fasting mimicking diets such as calorie restriction have been studied for their possible impact on autoimmune disease course (52). In Type 1 diabetes mellitus, caloric restriction was shown to improve glycemic control, downregulate inflammatory cytokines, such as interleukin 4, and IL-6, and upregulate anti-inflammatory cytokines as interleukin 10 (53). In MS, fasting mimicking diets were shown to attenuate symptoms by modulating immune cells and promoting oligodendrocyte regeneration (54).

## Immunity Changes in Fasting Patients with Cardiac Diseases

The effects of Ramadan fasting on the cardiac status of cardiac patients have not been clarified thus far. Khafaji et al. (39) investigated the possible relationship by examining the clinical progression, as well as leptin levels, and high sensitive (hs)-CRP levels before, during and after Ramadan in a sample of 56 patients (80.4% were male, 67.9% were aged >50 years) of different stable cardiac illnesses. 71.4% did not complain for any fluctuation of their symptoms, whereas 28.6% had improvement of their symptoms. Of note, higher compliance to medication occurred during Ramadan as compared to after. Moreover, 90% of the patients were compliant with diet during Ramadan with no significant changes in body weight. No cardiac or non-cardiac morbidity or mortality was reported. Biochemical levels of cholesterol, leptin and hs-CRP were not shown to significantly vary. Therefore, these results substantiate the safety of Ramadan fasting in cardiac patients with stable cardiac conditions. The effect of Ramadan fasting on cardiovascular risk factors was further investigated in a prospective observational study in a group of 82 patients (38 males and 44 females, aged 29–70 years, mean 54.0 ± 10 years) with at least one cardiovascular risk factor including coronary artery disease, metabolic syndrome, or cerebrovascular disease (CVD). Enrolled individuals were evaluated at least 10 h after fasting, before, and after Ramadan. Fasting samples were obtained including lipid profile, fasting blood sugar and insulin, hcy, hs-CRP and complete blood count; as well as blood pressure was measured and BMI was calculated. Fasting Ramadan led to a significant improvement of the 10-year coronary heart disease risk based on the Framingham risk score from the data collected above. No significant alterations were found in hs-CRP and hcy levels. These results point toward the usefulness of Ramadan fasting in improving CVD risk factors (40). Furthermore, in hypertensive patients, fasting has been shown to significantly reduce pulse pressure in the fasting hypertensive patients versus healthy controls. Additionally, MDA was shown to be significantly decreased, in contrast, GSH was significantly increased (38).

## Immunity Changes in Fasting Pregnant Women

Ozturk et al. (42) investigated the influence of Ramadan fasting during pregnancy on the maternal and the fetus status in a prospective controlled study. They enrolled 42 fasting and 30 non-fasting women in their second trimester of pregnancy. Total antioxidant status (TAS), total oxidant status (TOS), and the oxidative stress index (OSI) were determined from serum samples. Additionally, maternal complications, birth weight as well as other parameters were documented at the end of pregnancy.

The only significant finding was an elevated TAS in the fasting group; however, non-significant differences were documented in TOS and OSI. Their results showed that Ramadan fasting did not have a significant effect on the maternal oxidative stress, fetal development, maternal complications or fetal birth weight.

## Ramadan Fasting in Patients with Other Diseases

Among asthmatic patients, in a case–control study involving 15 asthmatic patients and 14 controls, fasting was shown to significantly decrease CRP levels. Pulmonary function tests levels post fasting were not shown to demonstrate any significant difference between both groups. Moreover, respiratory symptoms were not significantly increased. These results highlight the safety and lack of negative impact on asthma with some possible positive effects reflected by reduction of CRP levels (37).

In HIV patients on antiretroviral therapy, once-daily compared to twice daily dose therapy in fasting patients was not shown to lead to any significant changes in cluster differentiation 4 cell counts, viral load, or hematocrit levels (41).

In outpatient schizophrenic patients, a prospective study on 100 patients revealed that Ramadan fasting had a negative impact rather than a positive one on schizophrenic patients. This was reflected by a significant increase of lymphocytes, monocytes, fibrinogen and hs-CRP, especially in patients with metabolic syndrome. These results contrast with the many previously mentioned studies that reported the beneficial role of fasting on different inflammatory disease states (43).

## Ramadan Fasting in Athletes

The behavioral fluctuation that occurs during Ramadan including food and drink abstinence is thought to impact athletes on intensive regimens. The influence of intermittent fasting (Ramadan model) on the inflammatory and immunologic measures of 15 elite male judo athletes was monitored. Serum CRP increased from 2.93 ± 0.26 to 4.60 ± 0.51 mg/L, IgA increased from 1.87 ± 0.56 to 2.49 ± 0.75 g/L and persisted high for 3 weeks. Hcy remained relatively unchanged, as well as leukocyte counts, which also remained stable through the examined period. Such results highlight the various yet minimal fluctuations of immunologic markers that might occur in athletes who maintain a high intensity training program during Ramadan (49). In another studies, recreational bodybuilders did not show any significant changes in CRP levels (47). Among rugby players, IgA levels were demonstrated to be elevated as shown in previous studies (45).

Other Ramadan-associated behavioral fluctuations include the change of sleep habits, which might disturbs athletes’ physical performance. To study this effect, the performance of eight middle-distance athletes (average age of 25 years) was monitored 5 days before Ramadan, on the seventh day and on the 21st day of Ramadan fasting. Maximal aerobic velocity (MAV) test was performed on these days. Salivary cortisol, testosterone secretions and IL-6 levels, which play a role in sleep mediation, were measured. Compared with basal values, MAV test decreased significantly during Ramadan, while hormone levels did not show any significant deviations. Fatigue levels as measured by the Mood States questionnaire were also significantly increased, concomitant with a significant increase of IL-6, adrenaline and noradrenaline and a significant decrease of melatonin. Noteworthy, all these parameters trended back to normal ranges in 1 week after Ramadan. These finding supports the influence of Ramadan on athletes as witnessed by sleep disturbances, energy deficiency, and fatigue (27).

Strenuous exercise has been shown to influence myriad components of the immune system including neutrophils and natural killer (NK) cells. Interleukin 12 (IL-12), an immune-regulatory cytokine, is an avid NK cell activator as well an important inducer of pro-inflammatory factors secretion. During fasting, IL-12 was noted to be significantly decreased at 1 and 4 weeks in comparison to 3 weeks after Ramadan. The fluctuation of IL-12 levels has been attributed to changes in dietary intake as well as to sleep pattern alterations (48).

Intermittent exercise performed during Ramadan fasting has also been shown to enhance lipid profile and decrease inflammatory markers of cardiovascular health. In 15 healthy soccer players performing the yo-yo intermittent recovery test (YYIRT) during the evening hours while fasting, higher levels of high density lipoproteins and apolipoprotein A1 were documented, and a decrease of low density lipoprotein (LDL), apolipoprotein B, lipoprotein particles, hcy and hs-CRP. Therefore, fasting and exercise work synergistically to reduce body mass, improve lipid profile, and modulate the inflammatory status in athletes (46). In another study, 20 male soccer players with a mean age of 17.5 years demonstrated an increase in TAS during YYIRT (44). In another study, the effect of Ramadan fasting on 79 soccer players aged 16–19 years showed a decrease in leukocyte count and CRP. These effects have been thought to occur as a result of eating habit changes (51).

## Conclusion

**Figure 2:**
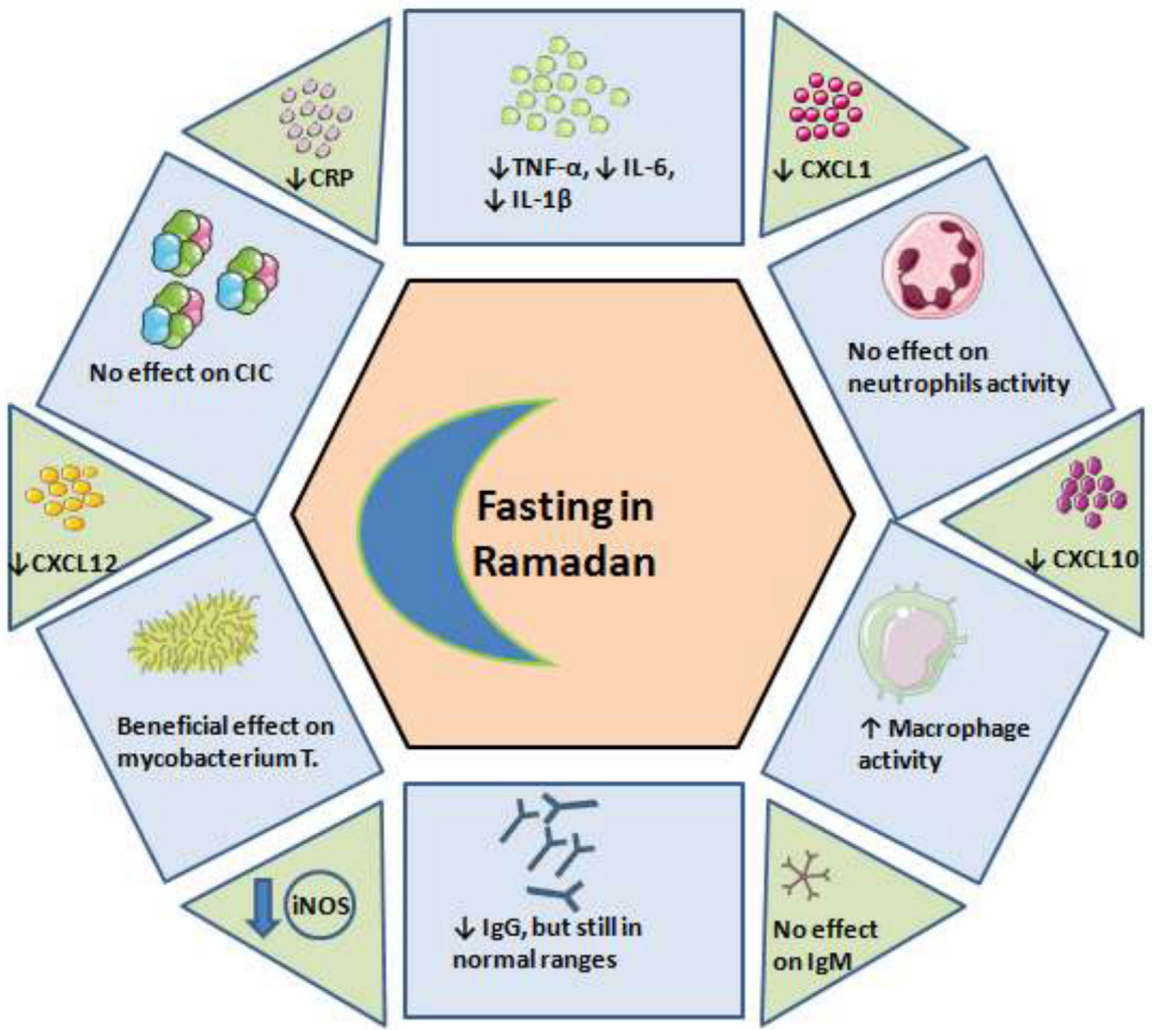
The effect of fasting on the immune system. CIC, circulating immune complexes; CRP, C-reactive protein; CXCL, chemokines; iNOS, inducible nitric oxide synthases.

Ramadan is observed by fasting in the Islamic communities; however, guidelines or consensus statements that help guiding physicians in addressing the issues of patients who are eager to fast are lacking. In this review, we aimed to systematically collect the current evidence concerning the influence of Ramadan fasting on the immune system. We can conclude that (Table 3; Figure 2):

1. Ramadan fasting has been shown to only mildly influence the immune system, and the alterations are transient and return to basal pre-Ramadan status.
2. Ramadan fasting during the second trimester of pregnancy was shown to be safe and did not result in negative fetal outcomes or maternal oxidative status alterations.
3. In cardiac patients, Ramadan fasting can have beneficial effects including lipid profile improvement and alleviation of oxidative stress.
4. In asthmatic patients, Ramadan fasting does not alter immunologic parameters.
5. In HIV patients, Ramadan was shown to be safe.
6. In patients with schizophrenia, Ramadan could increase immunologic markers.
7. In patients with autoimmune disorders, Ramadan was generally safe.
8. Fasting athletes who maintain intensive training schedule show fluctuations of immunologic markers.

## Author Contributions

NLB conceived and designed the study. MA, AW, NM wrote the manuscript. KA, HA, MZ, SB, KS, KG, RF, HM, SF, GSS critically revised the manuscript. All authors read and approved the final version of the manuscript.

## Conflict of Interest Statement

The authors declare that the research was conducted in the absence of any commercial or financial relationships that could be construed as a potential conflict of interest.
